# Supplementary material for: Multifunctional graphene heterogeneous nanochannel with voltage-tunable ion selectivity
Source: Nat Commun. 2022 Aug 19;13:4894. doi: 10.1038/s41467-022-32590-9 (PMC9391377; doi:10.1038/s41467-022-32590-9)
Supplement: Supplementary file 1 — Supplementary Information [file 41467_2022_32590_MOESM1_ESM.pdf]

Supplementary Information to "Multifunctional Graphene Heterogeneous Nanochannel with Voltage-Tunable Ion Selectivity"

Shihao Su,[1,2,6] Yifan Zhang,[1,2,6] Shengyuan Peng,[1,2] Linxin Guo,[1,2] Yong Liu,[1,2] Engang Fu,[1] Huijun Yao,[3,4] Jinlong Du,[5] Guanghua Du,[*,3,4] and Jianming Xue[*,1,2]

1*State Key Laboratory of Nuclear Physics and Technology, School of Physics, Peking University, Beijing 100871, P. R. China*

2*CAPT, HEDPS and IFSA, College of Engineering, Peking University, Beijing 100871, P. R. China*

3*Institute of Modern Physics, Chinese Academy of Sciences, Lanzhou 730000, P. R. China*

4*University of Chinese Academy of Sciences, Beijing 100049, P. R. China*

5*Electron Microscopy Laboratory, School of Physics, Peking University, Beijing 100871, P. R. China*

6*These authors contributed equally: Shihao Su, Yifan Zhang*

E-mail: gh_du@impcas.ac.cn; jmxue@pku.edu.cn

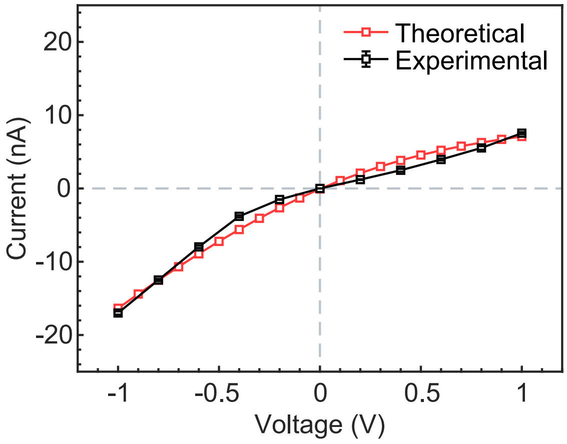

Supplementary Figure 1: Experimental and theoretical *I-V* curves for the pristine conical polyethylene terephthalate nanochannel (PETNC) in 0.1 M KCl solution. The theoretical results were obtained by numerically solving the Poisson-Nernst-Planck equations using COMSOL MultiPhysics Software for the PETNC with diameters of base and tip entrances respectively of 840 and 140 nm, and the surface charge density σ_{PET}=-0.08 C/m^2 which agrees with the value for a PETNC immersed in neutral electrolyte solutions in experiments.[1,2] The model of the PETNC was similar to that of the graphene-based polyethylene terephthalate nanochannel (GPETNC) without graphene in Supplementary Fig. 7. Theoretical models of PETNCs with different tip entrance diameters were constructed and their *I-V* curves in 0.1 M KCl solutions were calculated (data not shown) to estimate the tip entrance diameter of the PETNC in our experiments, which was ∼140 nm because a model with this size had the *I-V* curve best fitting to the experimental data.

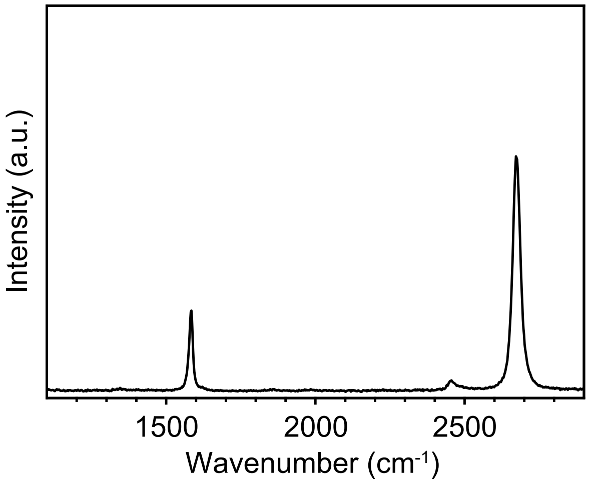

Supplementary Figure 2: Raman spectrum (532 nm excitation and 3 mW power) of pristine monolayer graphene synthesized using chemical vapor deposition method. There are a strong G peak at ∼1580 cm^{-1} and a negligible D peak at ∼1350 cm^{-1}, indicating the high quality of graphene with rare defects.[3,4] The intensity of the 2D peak at ∼2700 cm^{-1} (with a line width of ∼30 cm^{-1}) is about three times as strong as that of the G peak, which confirms that the graphene was monolayer.[3,4]

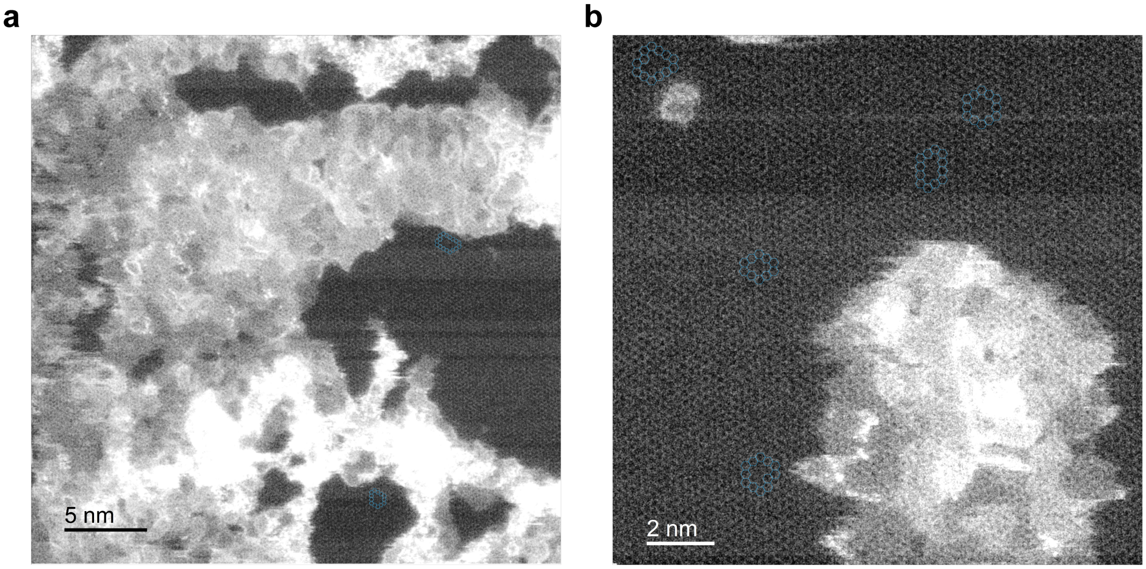

Supplementary Figure 3: Cs-corrected high-angle annular dark field-scanning transmission electron microscopy (HAADF-STEM) images of graphene with subnanopores fabricated under ion irradiation. **a**, **b** Two large graphene areas with atomic resolution. Seven recognizable subnanopores were found there and indicated by corresponding atomic structures, and four of them were exhibited in the manuscript as representatives. The numbers of missed carbon atoms in these subnaopores were 2, 4, 4, 5, 6, 7, and 13, and thus the areas of the pores could be respectively estimated to be 18, 36, 36, 45, 54, 64, and 118 Å^2 according to the carbon atom van der Waals diameter of 3.4 Å. Therefore, the average diameter of these pores (supposing they were circular) adjusted for van der Waals size of pore edge atoms[5] was calculated to be 5 Å, which agrees well with the prediction of our molecular dynamics simulations.[6] The total area of the images is 1280 nm^2, and thus the density of the graphene subnanopores was estimated to be 5.47×10^{11} cm^{-2}. In consideration of the incident ion fluence of 1×10^{13} cm^{-2}, the probability of generating a subnanopore by a single ion could be estimated to be ~5%, which is also consistent with the theoretical result of our previous work.[6]

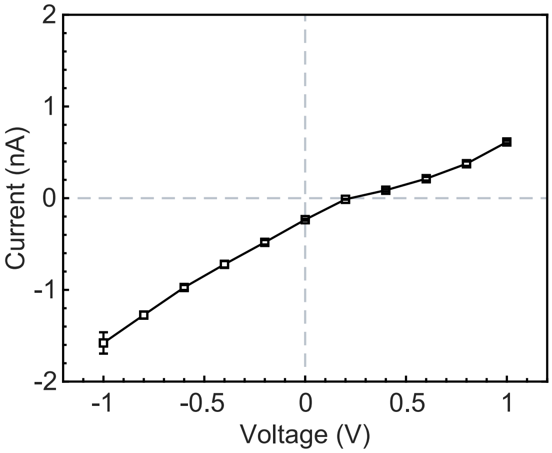

Supplementary Figure 4: *I*-*V* curve for the graphene-based polyethylene terephthalate nanochannel (GPETNC) in KCl solutions with concentration ratio of 1000:1 mM. The base side of the GPETNC had 1 mM KCl solution, where voltages were applied; while the tip side had 1000 mM KCl solution, where the electric potential was zero.

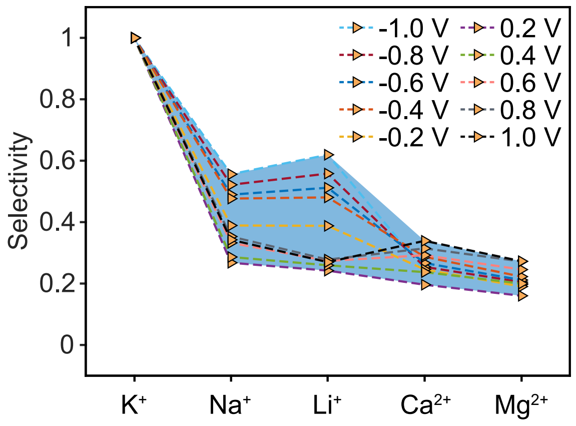

Supplementary Figure 5: Ion selectivity of the graphene-based polyethylene terephthalate nanochannel (GPETNC) at different voltages. The blue region is the range of tunable selectivity of the GPETNC in this work.

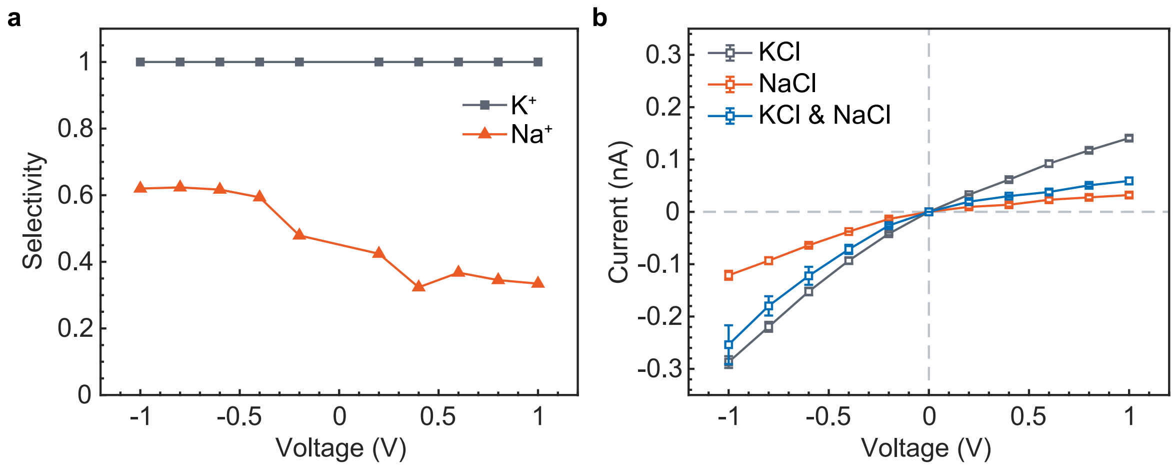

Supplementary Figure 6: Studies on graphene-based polyethylene terephthalate nanochannel (GPETNC) in mixed electrolyte solutions. **a** Ion selectivity of a GPETNC at different voltages. The presented results were from another GPETNC different from the one in the manuscript, which was fabricated to study the performance of GPETNC in mixed electrolyte solutions. **b** *I*-*V* curves of the GPETNC in (**a**) in 0.1 M KCl, 0.1 M NaCl, and 0.1 M KCl & 0.1 M NaCl solutions. Error bars were the standard deviations from the average of three independent records.

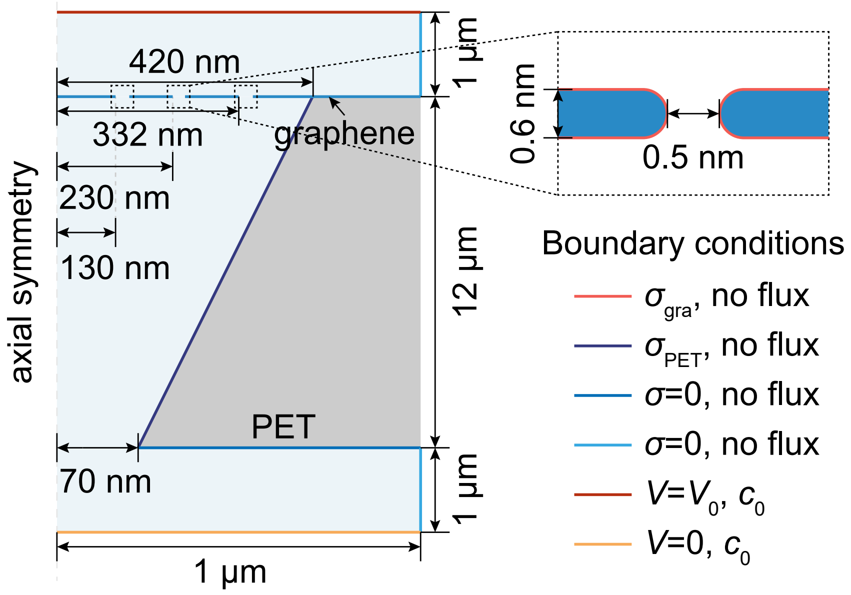

Supplementary Figure 7: Schematic illustration of the numerical model of the graphene-based polyethylene terephthalate nanochannel (GPETNC) in electrolyte solution using COMSOL MultiPhysics Software to solve the Poisson-Nernst-Planck equations. The diagram is not to scale for ease of view, and all the actual sizes are marked. The region to be solved is dyed in light blue, and corresponding boundary conditions are provided. σ is the surface charge density (different subscripts represent different boundaries), V is the electric potential (V_0 the applied voltage), and c_0 is the concentration of the electrolyte solution. σ_{PET} was set to be -0.08 C/m^2, in accordance with the value for a polyethylene terephthalate nanochannel (PETNC) immersed in neutral electrolyte solutions in experiments.[1,2] The nature of electric charges on nanoporous graphene remains elusive[7–12] for not only their uncertain positions (graphene surface due to hydroxide adsorption or pore edge due to functional groups), but also the inconsistent value of charge density (e.g., -0.2 C/m^2 in the work of Fu et al.,[11] -0.24 C/m^2 in the work of Shan et al.,[10] and -0.6 to -1 C/m^2 in the work of Rollings et al.[12]). Detailed studies of the origin of graphene charges and their influences on ion transport are beyond the scope of this work, and thus σ_{gra}=-0.05 C/m^2 was used here to improve the efficiency and convergence of calculations, and this value was reasonable because test simulations with σ_{gra}=-0.2 C/m^2 showed similar results. The model is two-dimensional axisymmetrical in consideration of computing resources, and therefore numerous subnanopores in graphene were modelled as several nano-annuluses (in three-dimension) with width of 0.5 nm, which is the same as the average diameter of graphene subnanopores observed in experiments (Supplementary Fig. 3) and predicted by computational simulations.[6] The number and location of these annuluses were carefully chosen to ensure that their total area in three-dimension equaled to the total area of graphene subnanopores of the GPETNC in experiments, which could be estimated to be 2176 nm^2 according to the diameter of the PETNC base entrance (840 nm), the fluence of incident ions (1×10^{13} cm^{-2}), the probability of generating a subnanopore by a single ion (~5%), and the average pore diameter (0.5 nm). For convenience, the modelled graphene nano-annuluses are nominally termed "subnanopores" in this work.

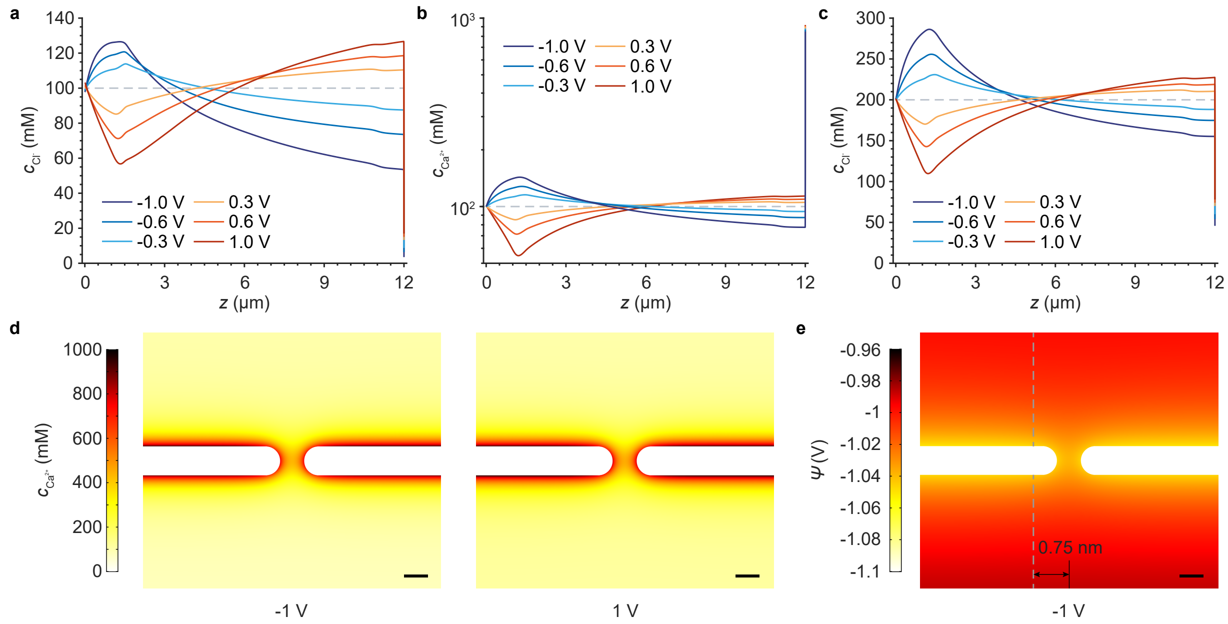

Supplementary Figure 8: Numerical calculations of distributions of ion concentration and electric potential in the graphene-based polyethylene terephthalate nanochannel (GPETNC). **a**-**c** Concentrations of chloride c_{Cl^-} (**a**), calcium $c_{Ca^{2+}}$ (**b**), and chloride c_{Cl^-} (**c**) ion along the center symmetry axis z of the GPETNC in 0.1 M KCl (**a**), $CaCl_2$ (**b**), and $CaCl_2$ (**c**) solutions at different applied voltages. z = 0 and 12 μm represent the tip and base entrance of the GPETNC, respectively. **d** Distributions of concentration of calcium ion $c_{Ca^{2+}}$ nearby a graphene subnanopore of the GPETNC in 0.1 M $CaCl_2$ solution at -1 and 1 V. Scale bars are 0.5 nm. **e** Distribution of electric potential ψ nearby a graphene subnanopore of the GPETNC in 0.1 M KCl solution at -1 V. Scale bar is 0.5 nm. The grey dashed line (with distance of 0.75 nm away from the pore center) is the path along which ψ is plotted in Fig. 4c in the manuscript.

References

1. Sheng, Q.; Wang, X.; Xie, Y.; Wang, C.; Xue, J. A Capacitive-Pulse Model for Nanoparticle Sensing by Single Conical Nanochannels. *Nanoscale* **2016**, *8*, 1565–1571.

2. Su, S.; Guo, X.; Fu, Y.; Xie, Y.; Wang, X.; Xue, J. Origin of Nonequilibrium 1/f Noise in Solid-State Nanopores. *Nanoscale* **2020**, *12*, 8975–8981.

3. Malard, L.; Pimenta, M.; Dresselhaus, G.; Dresselhaus, M. Raman Spectroscopy in Graphene. *Physics Reports* **2009**, *473*, 51–87.

4. Surwade, S. P.; Smirnov, S. N.; Vlassiouk, I. V.; Unocic, R. R.; Veith, G. M.; Dai, S.; Mahurin, S. M. Water Desalination using Nanoporous Single-Layer Graphene. *Nature Nanotechnology* **2015**, *10*, 459–464.

5. Wang, L.; Boutilier, M. S. H.; Kidambi, P. R.; Jang, D.; Hadjiconstantinou, N. G.; Karnik, R. Fundamental Transport Mechanisms, Fabrication and Potential Applications of Nanoporous Atomically Thin Membranes. *Nat. Nanotechnol.* **2017**, *12*, 509–522.

6. Su, S.; Xue, J. Facile Fabrication of Subnanopores in Graphene under Ion Irradiation: Molecular Dynamics Simulations. *ACS Applied Materials & Interfaces* **2021**, *13*, 12366–12374.

7. Ghosh, M.; Madauß, L.; Schleberger, M.; Lebius, H.; Benyagoub, A.; Wood, J. A.; Lammertink, R. G. H. Understanding Mono- and Bivalent Ion Selectivities of Nanoporous Graphene Using Ionic and Bi-ionic Potentials. *Langmuir* **2020**, *36*, 7400–7407.

8. Caglar, M.; Silkina, I.; Brown, B. T.; Thorneywork, A. L.; Burton, O. J.; Babenko, V.; Gilbert, S. M.; Zettl, A.; Hofmann, S.; Keyser, U. F. Tunable Anion-Selective Transport through Monolayer Graphene and Hexagonal Boron Nitride. *ACS Nano* **2020**, *14*, 2729–2738.

9. O'Hern, S. C.; Boutilier, M. S. H.; Idrobo, J.-C.; Song, Y.; Kong, J.; Laoui, T.; Atieh, M.; Karnik, R. Selective Ionic Transport through Tunable Subnanometer Pores in Single-Layer Graphene Membranes. *Nano Letters* **2014**, *14*, 1234–1241.

10. Shan, Y. P.; Tiwari, P. B.; Krishnakumar, P.; Vlassiouk, I.; Li, W. Z.; Wang, X. W.; Darici, Y.; Lindsay, S. M.; Wang, H. D.; Smirnov, S.; He, J. Surface Modification of Graphene Nanopores for Protein Translocation. *Nanotechnology* **2013**, *24*, 495102.

11. Fu, Y.; Su, S.; Zhang, N.; Wang, Y.; Guo, X.; Xue, J. Dehydration-Determined Ion Selectivity of Graphene Subnanopores. *ACS Applied Materials & Interfaces* **2020**, *12*, 24281–24288.

12. Rollings, R. C.; Kuan, A. T.; Golovchenko, J. A. Ion Selectivity of Graphene Nanopores. *Nature Communications* **2016**, *7*, 11408.
